# Reversal of ABCG2/BCRP-Mediated Multidrug Resistance by 5,3′,5′-Trihydroxy-3,6,7,4′-Tetramethoxyflavone Isolated from the Australian Desert Plant *Eremophila galeata* Chinnock

**DOI:** 10.3390/biom11101534

**Published:** 2021-10-18

**Authors:** Malene J. Petersen, Xamuel L. Lund, Susan J. Semple, Bevan Buirchell, Henrik Franzyk, Michael Gajhede, Kenneth T. Kongstad, Jan Stenvang, Dan Staerk

**Affiliations:** 1 Department of Drug Design and Pharmacology, Faculty of Health and Medical Sciences, University of Copenhagen, Universitetsparken 2, DK-2100 Copenhagen, Denmark; mjp@sund.ku.dk (M.J.P.); lund@cbs.cnrs.fr (X.L.L.); henrik.franzyk@sund.ku.dk (H.F.); mig@sund.ku.dk (M.G.); kenneth.kongstad@sund.ku.dk (K.T.K.); stenvang@sund.ku.dk (J.S.); 2 Centre de Biochimie Structurale (CBS), INSERM, CNRS, Université de Montpellier, 29 rue de Navacelles, 34090 Montpellier, France; 3 Quality Use of Medicines and Pharmacy Research Centre, Clinical and Health Sciences, University of South Australia, Adelaide, SA 5000, Australia; susan.semple@unisa.edu.au; 4 Wise Owl Consulting, Como, WA 6152, Australia; bevanbuirchell@gmail.com

**Keywords:** *Eremophila galeata*, breast cancer resistance protein, multidrug resistance, docking

## Abstract

Multidrug resistance (MDR) is a major challenge in cancer treatment, and the breast cancer resistance protein (BCRP) is an important target in the search for new MDR-reversing drugs. With the aim of discovering new potential BCRP inhibitors, the crude extract of leaves of *Eremophila galeata*, a plant endemic to Australia, was investigated for inhibitory activity of parental (HT29_par_) as well as BCRP-overexpressing HT29 colon cancer cells resistant to the chemotherapeutic SN-38 (i.e., HT29_SN38_ cells). This identified a fraction, eluted with 40% acetonitrile on a solid-phase extraction column, which showed weak growth-inhibitory activity on HT29_SN38_ cells when administered alone, but exhibited concentration-dependent growth inhibition when administered in combination with SN-38. The major constituent in this fraction was isolated and found to be 5,3′,5′-trihydroxy-3,6,7,4′-tetramethoxyflavone (**2**), which at a concentration of 25 μg/mL potentiated the growth-inhibitory activity of SN-38 to a degree comparable to that of the known BCRP inhibitor Ko143 at 1 μM. A dye accumulation experiment suggested that **2** inhibits BCRP, and docking studies showed that **2** binds to the same BCRP site as SN-38. These results indicate that **2** acts synergistically with SN-38, with **2** being a BCRP efflux pump inhibitor while SN-38 inhibits topoisomerase-1.

## 1. Introduction

Multidrug resistance (MDR) remains a major challenge in the treatment of patients suffering from different types of cancer. MDR is defined as resistance of cancer cells to structurally unrelated classes of chemotherapeutic drugs, and can manifest itself through various mechanisms. Reported mechanisms that appear to contribute to cancer MDR comprise, among others, the induction of apoptosis, hypoxia, autophagy, drug efflux, epigenetic regulation, and DNA damage/repair [1]. MDR can either be acquired during treatment or be pre-existing at the time of diagnosis, and it is a major cause of treatment failure in cancer patients [2], thus raising the need for drugs capable of reversing the resistance. 

Plasma membrane-bound transporters are highly involved in the uptake and/or efflux of chemotherapeutic drugs, and efflux pumps are very often associated with the development of MDR [3]. The ABC transporters are a family of transporter proteins (also termed ATP-binding cassette transporters), of which 12 out of the 48 known human transporters are drug transporters [1]. ABC transporters known to contribute to MDR are P-glycoprotein (P-gp/ABCB1), multidrug resistance protein 1 (MRP1/ABCC1), and breast cancer resistance protein (BCRP/ABCG2), which all are expressed in various tissues, playing important roles in drug transport and protection from toxins [4]. 

BCRP is a transmembrane protein consisting of 655 amino acids with one nucleotide-binding domain (NBD) and one membrane-spanning domain, and it is homodimerized in its functional state. The first structure of BCRP was determined by cryo-electron microscopy in 2017 [5], followed by structures of BCRP in the outward-facing ATP-bound conformation [6]. Structures of inhibitor-bound inward-facing BCRP are also available [7] and very recently the structure of BCRP binding SN-38 has been determined [8]. These studies have yielded detailed insight into the BCRP molecular mechanisms of substrate transport and inhibition. A wide range of compounds have been reported as substrates for BCRP, including different classes of drugs, conjugated organic anions, and fluorescent compounds, and the substrate specificity of BCRP and other drug-efflux transporters have been found to overlap. Chemotherapeutic drugs that are BCRP substrates include mitoxantrone, methotrexate, irinotecan, and SN-38, which complicates the treatment of, e.g., colorectal and breast cancer, where these specific drugs are commonly used. Fluorescent probes such as Hoechst 33342, pheophorbide A, and BODIPY-prazosin are also BCRP substrates that are useful for the study of BCRP function and activity [9].

Efflux pumps belonging to the ABC transporters constitute obvious targets in the search for MDR-reversing drugs, and several chemical substances have been investigated for their ability to modulate MDR through the inhibition of efflux pumps. First, second, and third-generation P-gp inhibitors have been developed, but currently no drugs exhibit significant MDR reversal without being toxic as well [6]. The first-generation inhibitors include verapamil, cyclosporine, and tamoxifen, whereas most of the second-generation inhibitors are analogues of the first-generation drugs, e.g., dexverapamil and valspodar. The third-generation inhibitors were developed based on QSAR analysis of the previous drugs, but so far, none of the drugs have been approved for the treatment of cancer patients [10]. Thus, there is a need for a new generation of drugs that are less toxic and more effective as compared to the existing drugs. 

Natural products have been proposed as a source of a fourth generation of MDR inhibitors [10,11], and several classes of compounds, e.g., flavonoids, alkaloids, coumarins, and terpenoids, have shown efflux pump-inhibitory activities [10]. Due to issues with toxicity among the existing efflux pump inhibitors, natural products constitute a promising source for new drug leads in the search for MDR modulators, as they possess high chemical diversity, promising bioactivity, and often low toxicity. 

*Eremophila* is a plant genus endemic to Australia, and it comprises more than 200 species. Secondary metabolites isolated from leaves of *Eremophila* species include several classes of terpenoids, lignans, fatty acids, verbascosides, and flavonoids [12,13,14], of which many have shown bioactivities. Thus, various *Eremophila* species have been shown to display antidiabetic [15,16,17], antiviral [18], antibacterial [19], cytotoxic [20], or anti-inflammatory [21] effects. However, several *Eremophila* species, including *Eremophila galeata* Chinnock, are incompletely investigated with respect to both phytochemistry and bioactivity. Several classes of natural products isolated from *Eremophila* species, e.g., flavonoids and terpenoids, have previously shown efflux pump-inhibitory activity. Therefore, the aim of this study was to investigate whether constituents of *E. galeata* were able to resensitize cancer cells resistant to the chemotherapeutic drug and BCRP substrate SN-38. 

## 2. Materials and Methods 

### 2.1. Chemicals and Reagents 

DMSO, Ko143, SN-38, Hoechst 33342, SDS, Crystal Violet solution, MTT [3-(4,5-dimethylthiazol-2-yl)-2,5-diphenyltetrazolium bromide], formic acid, HPLC-grade acetonitrile, and TPP^®^ tissue culture plates were purchased from Sigma-Aldrich/Merck (Darmstadt, Germany), and water was purified by deionization and 0.22 µm membrane filtration using a Millipore system (Billerica, MA, USA). Chloroform-*d* was purchased from Eurisotop (Gif-Sur-Yvette Cedex, France), and Roswell Park Memorial Institute (RPMI) 1640 GlutaMAX medium and foetal bovine serum (FBS) were purchased from Gibco (Gibco, Thermo Fisher Scientific, Waltham, MA, USA). 

### 2.2. Cell Lines and Culture Conditions 

Cell lines were maintained at 37 °C in a humidified 5% CO_2_ incubator. A parental HT29 cell line (HT29_par_) was obtained from the National Cancer Institute (NCI)/Development Therapeutics Program, and HT29par and its SN-38 resistant derivative, HT29_SN38_, were cultured in RPMI 1640-GlutaMAX medium supplemented with 10% FBS [22]. 

### 2.3. Extraction and Sample Preparation

*Eremophila galeata* Chinnock was collected in April 2018 by Dr. Bevan Buirchell 24.1 km south of Yalgoo on Paynes Find Road (28°30′46.7′′ S; 116°51′1.9′′ E). A voucher specimen was deposited at the herbarium of the University of Melbourne, Department of Botany (study voucher number EP245B, herbarium voucher number MELUD122731a). The plant material was frozen immediately following collection and then shipped on dry ice to the University of South Australia. The material was stored at −20 °C. Leaves (298.7 g) of *E*. *galeata* were submerged in 2.2 L of acetonitrile for 10 min to remove the leaf resin (not examined in this study), and subsequently the leaves were lyophilized with liquid nitrogen and crushed into a powder, which was extracted with 1.0 L of acetonitrile by shaking for 15 min using a Ratek Shaker (Ratek Instruments, Boronia, WI, Australia), and then filtered using a glass funnel. The crude filtrate was evaporated and freeze-dried to provide 11.54 g of dry extract, which was subsequently stored at −20 °C until further use. A sample of the dried crude extract (500 mg) was redissolved in 5% acetonitrile (*v*/*v*) to a final concentration of 125 mg/mL and subjected to solid phase extraction (SPE). Eight SPE fractions were eluted with 20 mL of 20%, 30%, 40%, 50%, 60%, 70%, 80%, and 100% acetonitrile (*v*/*v*), respectively. The procedure was repeated twice, giving fraction yields ranging from 1.5% to 21.7% of the total 500 mg. 

### 2.4. Analytical-Scale HPLC-PDA-HRMS 

All HPLC-PDA-HRMS analyses were performed by using an analytical-scale Agilent 1260 HPLC system (Agilent Technologies, Santa Clara, CA, USA), consisting of a G1329B autosampler, a G1311B quaternary pump with build-in degasser, a G1316A thermostatted column compartment and a G1316A photodiode array detector. Separations were performed at 30 °C on a Phenomenex Luna C18(2) reversed-phase column (150 × 4.6 mm i.d., 3 µm particle size, 100 Å pore size; Phenomenex, Torrance, CA, USA), with a flow rate of 0.5 mL/min. Eluent A (aqueous) consisted of water/acetonitrile (95:5, *v*/*v*), and eluent B (organic) of acetonitrile/water (95:5, *v*/*v*), both acidified with 0.1% formic acid. Compounds **1**–**3** were separated using the following gradient elution profile: 0 min, 20% B; 2 min, 25% B; 12 min, 35% B; 38 min, 50% B; 40 min, 100% B; 50 min, 100% B. The eluate was connected to a T-piece splitter directing 1% of the eluate into a Bruker micrOTOF-Q mass spectrometer equipped with an electrospray ionization (ESI) interface (Bruker Daltonik, Bremen, Germany). Mass spectra were acquired in positive ionization mode, using a drying temperature of 200 °C, a capillary voltage of 4100 V, a nebulizer pressure of 2.0 bar, and a drying gas flow of 7 L/min. Chromatographic separation and mass spectrometry were controlled by the Hystar ver. 3.2 software (Bruker Daltonik, Bremen, Germany).

### 2.5. Analytical-Scale HPLC-PDA and Fraction Collection 

A solution of 20 mg/mL of the SPE fraction eluted with 40% acetonitrile (*v*/*v*) was prepared in 40% acetonitrile:water (*v*/*v*). The solution was subjected to analytical-scale HPLC using an Agilent 1200 series instrument (Agilent Technologies, Santa Clara, CA, USA), consisting of a G1367C high-performance auto-sampler, a G1311A quaternary pump, a G1322A degasser, a G1316A thermostatted column compartment, a G1315C photodiode array detector and a G1364C fraction collector, all controlled by Agilent ChemStation version B.03.02 software. The 40% B fraction was separated on a reversed-phase Phenomenex Luna C18(2) column (150 × 4.6 mm i.d., 3 µm particle size, 100 Å pore size; Phenomenex, Torrance, CA, USA) using the same solvents and gradient elution profile as described in Section 2.5. Three peaks at retention times 22.6 min, 24.5 min and 27.6 min, respectively, were collected automatically from consecutive injections (20 µL per injection, flow rate 0.5 mL/min), and the fractions were dried overnight on a SPD121P Savant SpeedVac concentrator equipped with an OFP400 oil-free pump and a RVT400 refrigerated vapor trap (Thermo Fisher Scientific, Waltham, CA, USA). 

### 2.6. NMR Experiments 

The 1D ^1^H NMR spectra and proton-detected 2D NMR spectra were recorded on a 600 MHz Bruker Avance III HD instrument (operating at a proton frequency of 600.13 MHz), equipped with a 5 mm cryogenically cooled DCH probe (Bruker Biospin, Rheinstetten, Germany). All spectra were recorded at 298 K in chloroform-*d*, and ^1^H and ^13^C chemical shifts were referenced to the residual solvent signals at δ 7.26 ppm and 77.1 ppm, respectively. The ^1^H NMR spectra were recorded with a spectral width of 12 kHz, an acquisition time of 2.73 s, and a relaxation delay of 1.0 s, collecting 128 FIDs, each consisting of 64 k data points and Fourier transformed to 128 k data points with a line broadening factor of 0.3 Hz. The HSQC experiments were performed by collecting 64 FIDs in F2, each consisting of 1 k data points and corresponding to a spectral width of 7211.54 Hz. A total of 256 increments corresponding to a spectral width of 165 ppm were acquired to obtain the indirect dimension. The data were Fourier transformed and zero-filled to 4 k × 1 k data points (F2 × F1). HMBC experiments were performed by collecting 64 FIDs in F2, each consisting of 2k data points and corresponding to a spectral width of 7211.54 Hz. A total of 256 increments corresponding to a spectral width of 12 ppm were acquired to obtain the indirect dimension. The data were Fourier transformed and zero-filled to 4 k × 1 k data points (F2 × F1). IconNMR ver. 4.2 (Bruker Biospin, Rheinstetten, Germany) was used for controlling data acquisition and Topspin ver. 3.6.0 (Bruker Biospin, Rheinstetten, Germany) was used for acquisition and processing of NMR data.

### 2.7. MTT Cell Viability Assay 

For the seven *E*. *galeata* SPE fractions, three concentrations of each extract fraction were prepared from a 50 mg/mL stock solution in DMSO. Concentrations of 25, 12.5, and 6.125 µg/mL were prepared by serial dilutions with RPMI 1640-GlutaMAX medium (≤0.8% DMSO). HT29_SN38_ or HT29_par_ cells were seeded into 96-well microplates with a density of 8000 cells/well. Following overnight incubation (37 °C, 5% CO_2_), 100 µL of each SPE fraction (with the above concentrations) or 1 µM Ko143 were added to the cells in duplicates, either alone or together with 0.05 and/or 0.005 µM SN-38. Plates were incubated for 72 h, and then 3-(4,5-dimethylthiazol-2-yl)-2,5-diphenyltetrazolium bromide (MTT) reagent (5 mg/mL in PBS (*w*/*v*) diluted 1:10 in medium (*v*/*v*) reaching a final concentration of 0.5 mg/mL) was subsequently added to each well. MTT stop buffer (20% SDS in 0.02 M HCl) was added after 3 h of incubation (37 °C, 5% CO_2_), and then plates were incubated for approximately 3 h to facilitate dissolution of formazan crystals. Experiments were performed in three biological replicates. The absorbance at 570 nm and 670 nm was measured for each well by using a PowerWaveX^TM^ Select microplate spectrophotometer (BioTek, Winooski, VT, USA), and the background (670 nm) was subtracted prior to data analysis. The percentage of growth inhibition was calculated according to Equation (1):

(1) $$\%\;growth\;inhibition=100-\left(\left(\frac{OD_{sample}}{OD_{blank}}\right)\times 100\%\right)$$

where *OD_sample_* contains medium, cells, and test compound(s) (SN-38, Ko143, fractions and/or isolated compounds **1**–**3**) and *OD_blank_* contains medium and cells.

### 2.8. Dye Accumulation Assay

HT29_par_ and HT29_SN38_ cell lines were seeded into 96-well microplates with a density of 8000 cells/well and incubated at 37 °C (5% CO_2_) for approximately 24 h. The cells were subsequently treated with triplicates of 0.78, 1.56, 3.13, 6.25, 12.5, 25, 50, and 75 µg/mL of compound (**2**) or 0.001, 0.01, 0.025, 0.05, 0.1, 0.25, 0.5, and 1 µM Ko143, followed by incubation for 1 h (37 °C, 5% CO_2_). The fluorescent dye, Hoechst 33342 (5.0 µg/mL), was added to each well and to a blank control well (all in triplicates), and the plates were subsequently incubated for 30 min (37 °C, 5% CO_2_). After incubation, cells were washed with ice-cold PBS while being kept on ice, and subsequently wrapped in aluminium foil prior to further analysis. The experiments were performed in three biological replicates. The fluorescence intensity at excitation/emission wavelengths of 346/460 nm was measured for each well by using a SpectraMax i3x microplate reader (Molecular Devices, San Jose, CA, USA). The results were used for determining IC_50_ values in GraphPad Prism software, version 7.03 (GraphPad software, San Diego, CA, USA). Data were fitted to Equation (2):

(2) $$f\left(x\right)=\min+\frac{\max-min}{1+{\left(\frac{x}{IC_{50}}\right)}^{slope}}$$

where min is the background, max − *min* is the y-range, *x* is the concentration and slope is the Hill slope. Results are reported as IC_50_ values ± standard error.

### 2.9. Colony Formation Assay

The effect of **2** (1.25, 2.5, 5.0, or 10.0 µg/mL) either alone or in combination with SN-38 (10 nM) on the colony formation capacity of HT29_SN-38_ and HT29_Par_ cells was evaluated by a colony formation assay [23]. Cells were plated overnight into 12-well plates (200 cells/mL for HT29_Par_ and 800 cells/mL for HT29_SN-38_), followed by treatment with **2** and SN-38, either in combination or alone, respectively. Ko143 (0.5 µM) was used as a positive control for ABCG2-dependent resensitization of the resistant cells to SN-38. The cells were incubated at 37 °C in a humidified 5% CO_2_ incubator for 7 days, or until a sufficient amount of colonies had formed in the control wells. After incubation, the cells were stained with Crystal Violet solution and the colonies were counted using Image J software. The surviving fraction (SF) was determined using the formula SF = (no. of colonies formed after treatment)/(no. of cells seeded × plating efficiency). 

### 2.10. Statistical Analyses 

Statistical analyses were performed using GraphPad Prism software, version 9.01 (GraphPad software, San Diego, CA, USA). For data expressed in percentages, the data were expressed as a mean with standard deviations and significant differences were determined by using the multiple comparison unpaired *t*-test (Holm-Sidak method) where relevant. The significance level was set to 5%, and *p*-values less than 0.05 were considered significant. Statistical significance were shown on the graphs using the p value classification system, expressed as * *p* ≤ 0.05, ** *p* ≤ 0.01, *** *p* ≤ 0.001, **** *p* ≤ 0.0001.

### 2.11. Molecular Interaction Modelling 

The interaction between compound **2** and the BCRP transporter was studied by docking the ligand using Glide, Schrödinger Release 2019-3 LLC [24,25,26]. Using the Protein Preparation Wizard, Schrödinger 2019-3, LLC [27], the 3D structure of BCRP was built from the cryo-EM structure of the BCRP (PDBID: 6ETI) [7]. Compound **2** was docked by using extra precision (XP) flexible docking while allowing the sampling of ring conformations and nitrogen inversions. The docking environment was previously used to investigate other possible inhibitors for BCRP [28]. The ligand was constructed by using Ligprep, Schrödinger 2019-3, LLC.

Molecular dynamics simulations were performed to characterize the interactions between compound **2** and BCRP. The simulations were performed using Desmond Molecular Dynamics System, D.E. Shaw Research, Schrödinger 2020-1, LLC [26]. Compound **2** docked in BCRP was prepared by fitting a standard lipid membrane to the membrane-spanning domain followed by saturation of the system with ions and water molecules. The molecular dynamics simulation was run for six runs of 100 ns. The simulated trajectories were merged into one 600 ns trajectory and analysed by using the Simulations Interactions Diagram, Desmond, Schrödinger 2019-3, LLC [29].

## 3. Results

### 3.1. The Effect of SN-38 Alone and in Combination with BCRP Inhibitor Ko143 on HT29 Colon Cancer Cells

**Figure 1:**
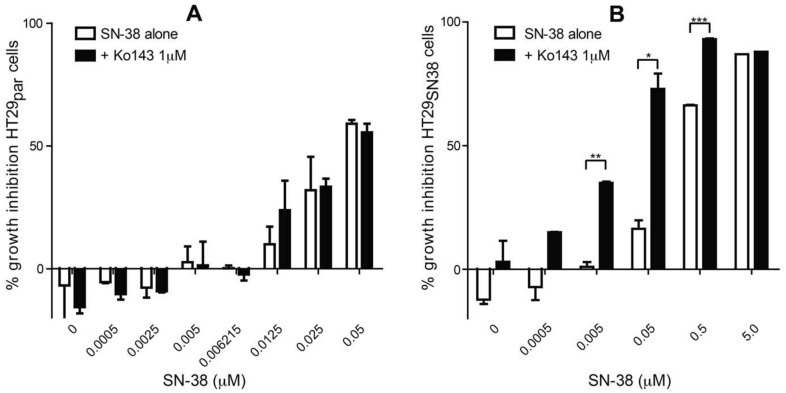
SN-38 dose–response experiments on HT29 parental (HT29_par_, ((**A**) and HT29 SN-38 resistant (HT29_SN38_, ((**B**) cell lines. Growth inhibition is expressed as percentage relative to untreated control. Error bars indicate SD determined on the basis of *n* = 2. * *p* ≤ 0.05, ** *p* ≤ 0.01, *** *p* ≤ 0.001.

To assess possible synergistic effects between SPE fractions of *E. galeata* and SN-38 (an antineoplastic drug acting as a topoisomerase 1 inhibitor), an assay was set up to measure increases in growth inhibition when combining SN-38 and SPE fractions. To validate this assay, growth inhibition was measured with different concentrations of SN-38 combined with 1.0 µM of the reference ABCG2/BCRP inhibitor Ko143. The assay was performed with a parental HT29 colon cancer cell line (HT29_par_) and a HT29 colon cancer cell line made resistant to SN-38 (HT29_SN38_), of which the latter has previously been shown to be upregulated in the ABCG2 gene and to be overexpressing ABCG2/BCRP [22]. A dose-response experiment was performed on HT29_par_ (Figure 1A) and HT29_SN38_ cells (Figure 1B), to confirm SN-38 resistance as well as to identify the SN-38 concentration at which the cells were minimally affected by SN-38 alone.

As observed in Figure 1B (white bars), the HT29_SN38_ cells needed a higher SN-38 concentration to reach the same level of inhibition of cell growth as in HT29_par_ (Figure 1A, white bars), in line with an expected increased efflux of the topoisomerase 1 inhibitor SN-38 due to the previously shown upregulation of BCRP in this cell line [22]. Furthermore, it was observed that the BCRP inhibitor Ko143 was able to resensitize HT29_SN38_ cells toward SN-38 without being cytotoxic itself (Figure 1B, black bars), whereas this effect was not seen in HT29_par_ (Figure 1A). Thus, for concentrations of 0.005, 0.05, and 0.5 μM of SN-38, the growth inhibition of HT29_SN38_ cells was significantly higher when tested alone compared to when tested together with 1 μM of the ABCG2/BCRP inhibitor Ko143. Therefore, these results confirmed that the HT29_SN38_ cells had acquired resistance toward SN-38, and that an ABCG2 inhibitor could selectively restore sensitivity to SN-38 in HT29_SN38_ cells overexpressing ABCG2, whereas this was not the case in the HT29_par_ cells that do not express significant levels of ABCG2. SN-38 concentrations of 0.005 µM and 0.05 µM were used in further synergy experiments for the HT29_par_ and HT29_SN38_ cell lines, respectively. The concentration of 0.05 µM was chosen since the increase in growth inhibition, when combined with 1.0 µM Ko143 (=0.470 μg/mL Ko143), was highest at this concentration. The parental cell line was used as a control, to show that the same synergistic effect is not present in cells that are not resistant to SN-38. 

### 3.2. Screening of SPE Fractions

**Figure 2:**
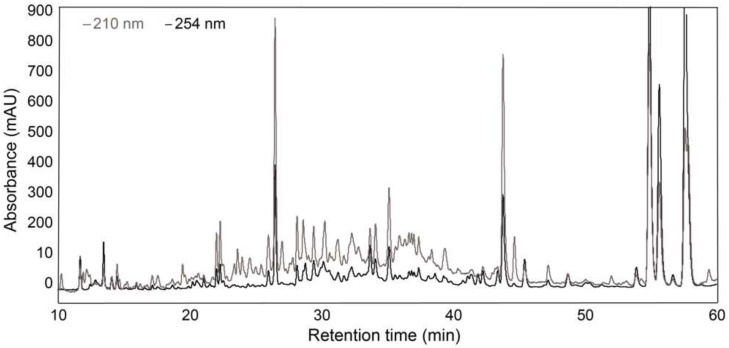
HPLC-PDA chromatogram of the crude acetonitrile extract of *E. galeata* (grey: 210 nm, black: 254 nm).

*E. galeata* is a species not previously assessed for bioactivity. However, various species of the *Eremophila* genus have been found to exert several bioactivities, and compound classes known to have efflux pump-inhibitory potential have previously been isolated from this genus as well. It was therefore decided to screen the *E. galeata* extract for potential synergistic effect with SN-38. Leaves of *E. galeata* were extracted with acetonitrile, and the chemical profile was assessed via its HPLC-PDA chromatogram. The crude extract exhibited a very complex chemical profile (Figure 2), and it was therefore decided to fractionate the crude extract further by using solid-phase extraction (SPE) to allow for an easier pinpointing of constituents correlated with bioactivity.

**Figure 3:**
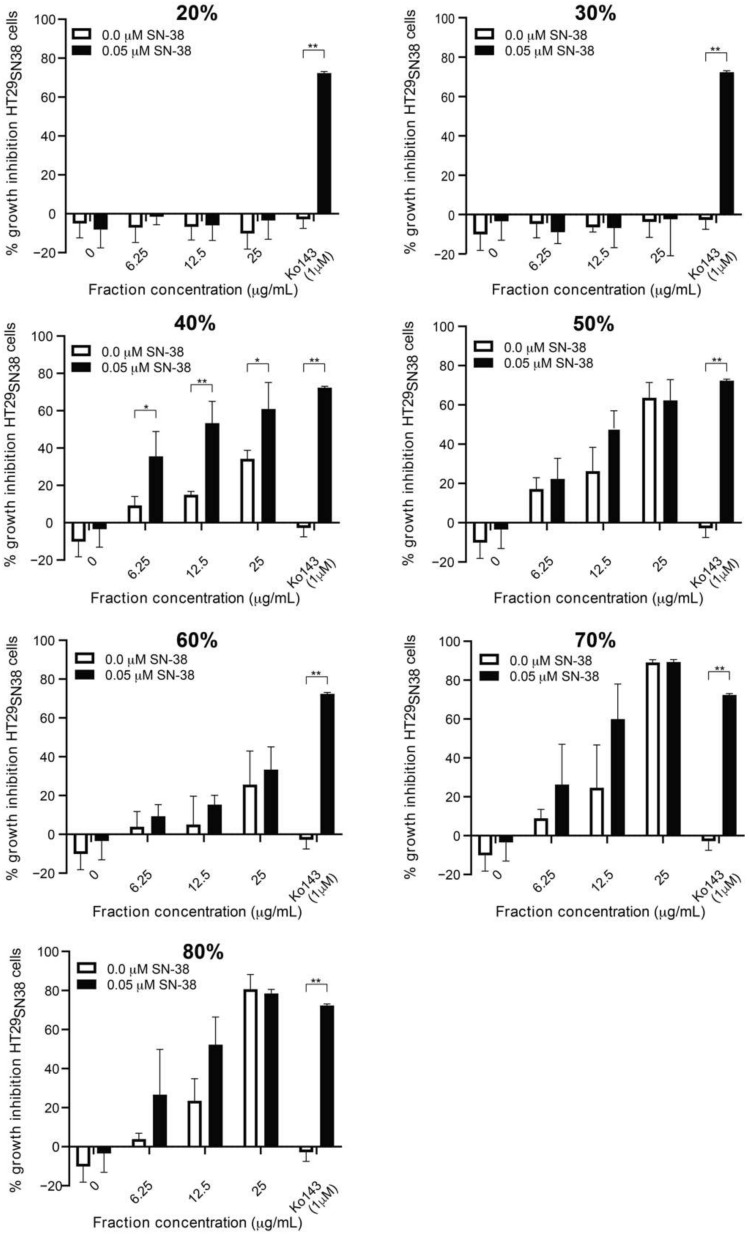
Growth inhibition (relative to untreated controls) of HT29 SN-38 resistant cells treated with SPE fractions eluted with 20%, 30%, 40%, 50%, 60%, 70%, and 80% acetonitrile:water, either alone or in combination with 0.05 µM SN-38. Ko143 (at 1 µM) was used as a positive control. Error bars indicate SD determined on the basis of *n* = 4. * *p* ≤ 0.05, ** *p* ≤ 0.01.

The crude extract was fractionated into eight SPE fractions, eluted with 20–100% acetonitrile:water (*v*/*v*), and seven of these fractions were subsequently screened for synergy with SN-38. The potential synergistic activity was investigated by adding 0.05 µM SN-38 together with different concentrations of each fraction, followed by a cell viability assay to assess whether any of the fractions could increase the effect of SN-38 without showing growth-inhibitory effects by themselves. As seen in Figure 3, the fractions eluted with 20% and 30% acetonitrile did not show any growth inhibition, either alone or when combined with SN-38, and therefore were these fractions not investigated further. Furthermore, it was observed that the fractions eluted with 50%, 60%, 70%, and 80% acetonitrile showed a dose-dependent increase in growth inhibition, which showed no significant difference whether tested alone or in combination with 0.05 μM SN-38. It was therefore concluded that the observed effects of these SPE fractions were a result of these fractions’ growth-inhibitory activity and not arising from synergy with SN-38, and the 50%, 60%, 70%, and 80% fractions were not included in further investigations. The fraction eluted with 40% acetonitrile showed the most promising effect, since the growth inhibition increased significantly in a dose-dependent manner when this fraction was tested together with 0.05 µM SN-38 (black bars) as compared to testing the extract with no SN-38 added (white bars). This indicated a synergistic effect of the 40% SPE fraction with SN-38 on the HT29_SN38_ cell line.

### 3.3. Further Testing of the Most Active Fraction 

The above screening of the seven different SPE fractions using the HT29_SN38_ cell line showed that the fraction eluted with 40% acetonitrile exhibited the highest, and the only statistically significant, increase in growth inhibition when tested together with 0.05 µM SN-38 as compared to the fraction’s own very low growth-inhibitory activity. Therefore, the 40% fraction was further tested for the ability to resensitize HT29_SN38_, and the effect on HT29_par_ was evaluated as well. During all experiments, the positive control Ko143 was included, to confirm the functionality of the assay and the importance of ABCG2 in the resensitization to SN-38.

**Figure 4:**
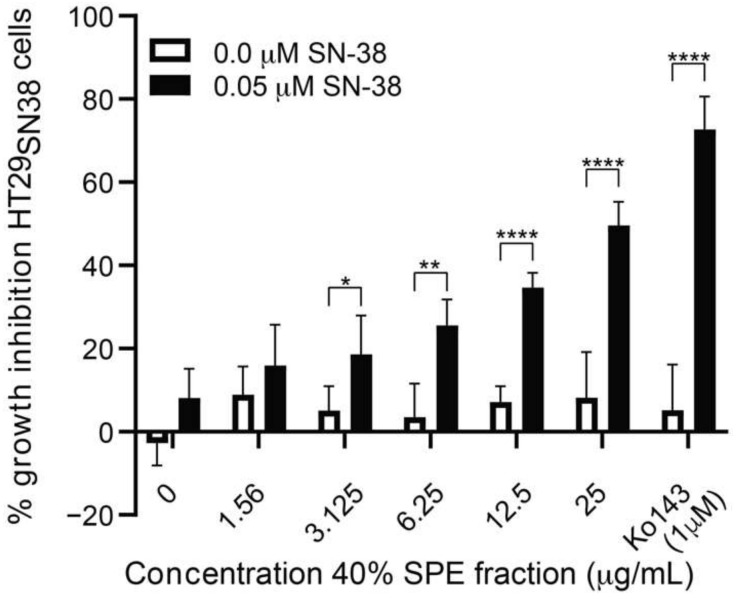
Growth inhibition (relative to untreated controls) of HT29_SN38_ cells treated with different concentrations of 40% SPE fraction, either alone or in combination with 0.05 µM SN-38. Ko143 at 1 µM was used as a positive control (duplicates performed in 3 biological replicates). Error bars indicate SD determined on the basis of *n* = 6. * *p* ≤ 0.05, ** *p* ≤ 0.01, **** *p* ≤ 0.0001.

From the white bars of the growth inhibition curve in Figure 4, it can be seen that the 40% fraction without SN-38 added showed a quite constant growth inhibition of HT29_SN38_ cells around 10%, i.e., showing that this fraction has almost no growth-inhibitory activity up to a concentration of 25 μg/mL. However, as seen from the black bars of Figure 4, the presence of 0.05 µM SN-38 results in a concentration-dependent increase in growth inhibition from 8.0 ± 2.9% at 0 µg/mL of the 40% SPE fraction (SN-38 alone) to 49.60 ± 2.31% at a concentration of 25 µg/mL of the 40% SPE fraction. This indicates that one or more compounds from this fraction most likely increased the effect of SN-38 on HT29_SN38_ cells.

### 3.4. Isolation and Test of Pure Compounds from the 40% Acetonitrile Fraction

**Figure 5:**
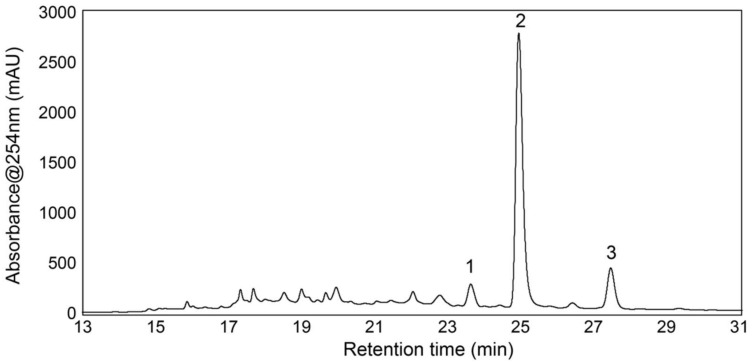
HPLC chromatogram at 254 nm of the 40% acetonitrile fraction.

The chemical profile of the SPE fraction eluted with 40% acetonitrile was further investigated, and as can be seen in Figure 5, the fraction showed a simple HPLC chromatogram with only a few peaks. 

**Figure 6:**
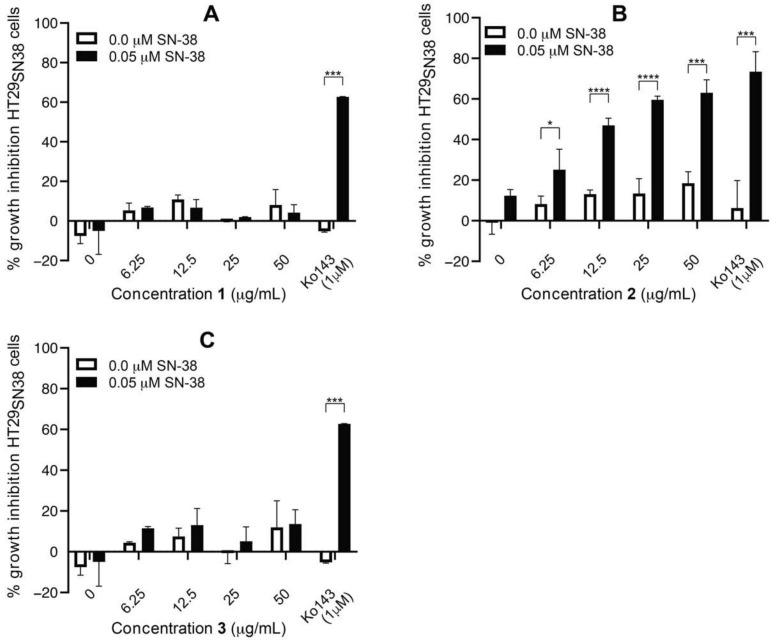
Percent cell-growth inhibition of HT29_SN38_ cells treated with 6.25, 12.5, 25, and 50 µg/mL of compounds **1** (**A**), **2** (**B**), and **3** (**C**) alone and in combination with 0.05 µM SN-38. Ko143 (1µM) was used as a positive control. Error bars indicate SD determined on the basis of *n* = 2. * *p* ≤ 0.05, *** *p* ≤ 0.001, **** *p* ≤ 0.0001.

The material eluted with peaks one, two, and three were isolated (and denoted compounds **1**–**3**) and tested for synergistic effects with SN-38 on HT29_SN38_ cells, as detailed in experimental Section 2.4. As can be seen from Figure 6A,C, compounds **1** and **3** did not show a growth-inhibitory effect at any of the tested concentrations, either alone or in combination with 0.05 µM SN-38. Figure 6B shows that compound **2** also did not possess any significant growth-inhibitory activity when tested alone. However, when **2** was tested in combination with 0.05 µM SN-38, the growth-inhibitory activity increased in a concentration-dependent manner from 12% at 0 µg/mL of **2** (SN-38 alone) to 63% at 50 µg/mL of **2**. 

For all tested concentrations of **2**, the growth-inhibitory activity was significantly higher with SN-38 added than without SN-38 (*p* values ranging from 0.05 to 0.0001). This indicated that **2** was able to increase the effect of SN-38 on HT29_SN38_ cells synergistically.

**Figure 7:**
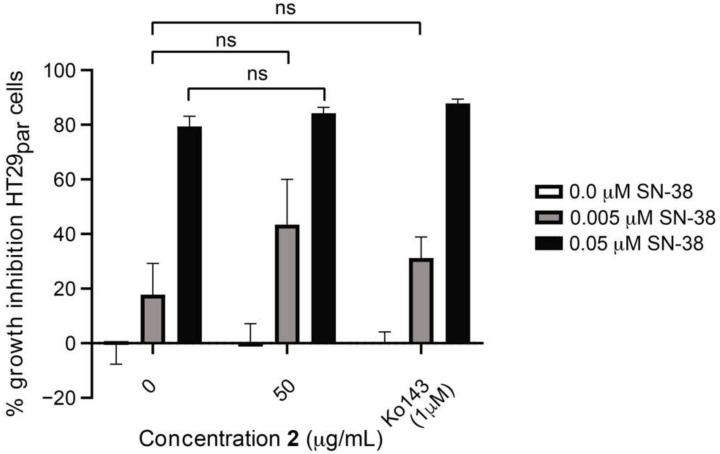
Percent cell-growth inhibition of HT29_par_ cells treated with 50 µg/mL of compound **2** alone and in combination with 0.005 or 0.05 µM SN-38. Ko143 (1 µM) was used as a positive control. Error bars indicate SD determined on the basis of *n* = 4. ns = not statistically significant.

In Figure 7, the growth-inhibitory activity of **2** and Ko143 on the HT29 parental cell line HT29_par_, either alone or combined with 0.005 µM or 0.05 µM SN-38, is shown. Thus, at both 0.005 µM and 0.05 µM of SN-38, no significant differences in growth inhibition are observed between untreated cells and cells treated with either **2** or Ko143. Thus, the growth inhibition seen must be an effect of SN-38, and not the added compounds, which is consistent with the fact that HT29_Par_ is SN-38-sensitive. The results therefore indicate that **2** does not increase the effect of SN-38 on HT29_par_, which suggests that **2** exerts its effect via ABCG2/BCRP modulation, since this efflux pump is overexpressed in the HT29_SN38_ cells, but not in HT29_par_ cells [22].

Compound **2** was, as seen in the chromatogram in Figure 5, the major peak in the 40% acetonitrile SPE fraction, and it was thus expected, even though the fraction contained other minor compounds, that **2** alone was responsible for the activity seen for this fraction (Figure 4). 

**Figure 8:**
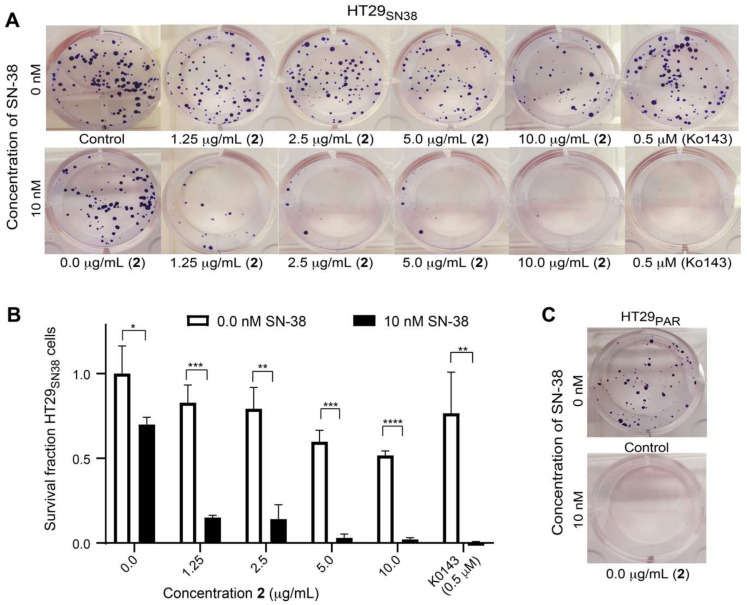
Effect of **2** (1.25, 2.5, 5.0 or 10 µg/mL) and Ko143 (0.5 µM) on the colony formation capacity of HT29_SN38_ cells, either alone or in combination with 10 nM SN-38 (**A**). Survival fraction (SF) of HT29_SN38_ cells when treated with **2** alone or in combination with 10 nM SN-38. Untreated cells were used as controls, (**B**). Colony formation capacity of HT29_PAR_ untreated cells and cells treated with 10 nM SN-38 (**C**). Data expressed as mean ± SD of *n* = 3 data points. The data are representative for experiments obtained in three biological replicates. * *p* ≤ 0.05, ** *p* ≤ 0.01, *** *p* ≤ 0.001, ****, *p* ≤ 0.0001.

To further verify the synergistic effects of **2** with SN-38 on HT29_SN38_ cells, a colony formation assay was performed. The cells were seeded at a low density, and subsequently treated with either 1.25, 2.5, 5.0 or 10 µg/mL of **2** alone, in combination with 10 nM SN-38 or with SN-38 alone (10 nM) (Figure 8A). Ko143 (0.5 µM) was used as a positive control for ABCG2 inhibition and prevented colony formation in the presence of SN-38 (Figure 8A). The cells were incubated until a sufficient number of colonies had formed in the control wells, and then the colonies were counted and the survival fraction was calculated according to [23]. As can be seen in Figure 8B, the survival fraction (SF) of the HT29_SN38_ cells significantly decreased with increasing concentrations of **2** when also treated with SN-38, as compared to the SF of the cells treated with **2** alone. The fact that the SF is significantly reduced in cells treated with both **2** and SN-38, as compared to cells only treated with **2**, clearly shows that **2** increased the effect of SN-38 on the SN-38 resistant cells, suggesting synergistic effects between **2** and SN-38. This is further supported by the SF of the cells treated with SN-38 alone, which is not significantly reduced as compared to the control cells, showing that the effect is only present when treating with a combination of SN-38 and **2**. The non-resistant HT29_PAR_ cells did not form any colonies after exposure to 10 nM SN-38 (Figure 8C), confirming that the HT29_SN38_ cells are resistant toward SN-38. 

### 3.5. Structure Elucidation 

HPLC-PDA-HRMS, 1D and 2D NMR data were acquired to elucidate the structure of **2**. HPLC-PDA-HRMS data revealed that **2** had an *m*/*z* ratio of 391.1028, [M+H]^+^ (calcd 391.1024, ∆M −1.1 ppm), and thus a molecular formula of C_19_H_18_O_9_ with UV maxima of 219 nm, 272 nm, and 335 nm. The ^1^H NMR data showed that **2** contained four OMe groups and three singlet protons, of which two corresponded to a signal at 7.32 ppm. The yellow appearance of the compound together with the high number of oxygen atoms, the presence of a carbonyl carbon and a 15-carbon skeleton suggested **2** to be a flavone. When the literature was examined, a large variety of tetra-methoxylated flavones with a molecular formula of C_19_H_18_O_9_ were found. The fact that all protons were singlets justifies the assumption that the two equivalent protons at 7.32 ppm and the proton at 6.50 ppm are located in different rings, and that the protons at 7.32 ppm are positioned in a similar chemical environment. It could furthermore be concluded that none of the OMe groups are equivalent and thus are positioned in different chemical environments. Based on these criteria and a comparison with previously published data, compound **2** was identified as 5,3′,5′-trihydroxy-3,6,7,4′-tetramethoxyflavone (Table 1), which is also in agreement with previously published UV maxima and MS data for this compound [30]. 

No previously published NMR data of 5,3′,5′-trihydroxy-3,6,7,4′-tetramethoxyflavone were found, but the NMR data of **2** were in agreement with previously published NMR data of a flavone with an identical C5-C10 A-ring structure [31] and with that of 2-methoxybenzene-1,3-diol with an identical B-ring structure [32], further confirming the structure of **2**. 

### 3.6. Efflux-Pump Inhibition Study on Isolated Compound

To support that the synergistic effects seen between **2** and SN-38 in fact were a result of the inhibition of the BCRP efflux pump, a dye accumulation study was performed; here the fluorescent dye Hoechst-33342 (H33342), which also is a substrate of BCRP, was used.

**Figure 9:**
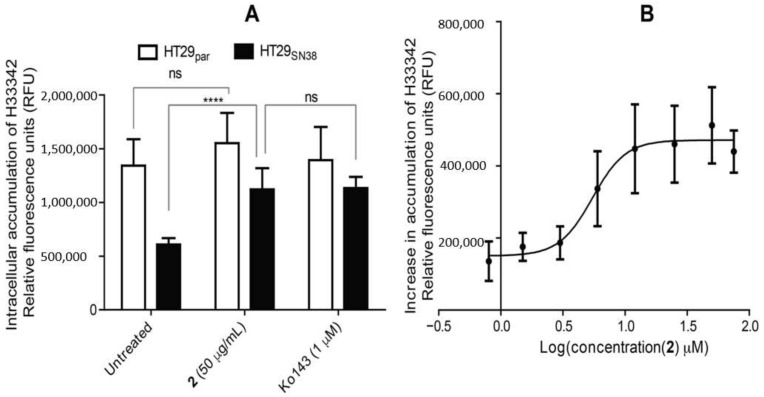
(**A**) Intracellular accumulation of H33342, expressed as relative fluorescence units (RFU) with excitation/emission 346/460, in untreated cells and cells treated with **2** or Ko143, all in the presence of 5 µg/mL H33342. Black: HT29_SN38_, white: HT29_par_. Error bars indicate SD determined on the basis of *n* = 6. (**B**) Dose–response curve for compound **2** with increase in accumulation of H33342 in HT29_SN38_ (relative to untreated control cells) measured as RFUs. Data for the dose–response curve are plotted as the mean of the mean of three biological replicates, and error bars indicate SEM with *n* = 3. **** *p* ≤ 0.0001, ns = not statistically significant.

The resistant HT29_SN38_ and non-resistant HT29_par_ cells were pre-treated with 50 µg/mL of **2** or 1 μM of Ko143, and then 5 µg/mL H33342 was added to each well followed by a second incubation. After rinsing the remaining media off, the relative fluorescence units (RFU) were measured to quantify the amount of intracellular accumulation of H33342. As seen in Figure 9A, intracellular H33342 (expressed as RFU) in untreated cells was higher for HT29_par_ as compared to the level for HT29_SN38_, confirming that resistant cells show an increased efflux of H33342, likely due to the overexpression of BCRP. Furthermore, it is seen in the HT29_SN38_ cells that **2** was able to increase the intracellular accumulation of H33342 as compared to untreated cells (*p* value 0.0001), and to a level comparable to the positive control, Ko143, strongly suggesting that **2** is a BCRP inhibitor. The IC_50_ value, expressed as increase in H33342 intracellular accumulation (as shown in Figure 9B) was determined to be 4.88 ± 1.32 µg/mL (14.59 ± 5.29 µM). 

### 3.7. Docking Studies on BCRP 

**Figure 10:**
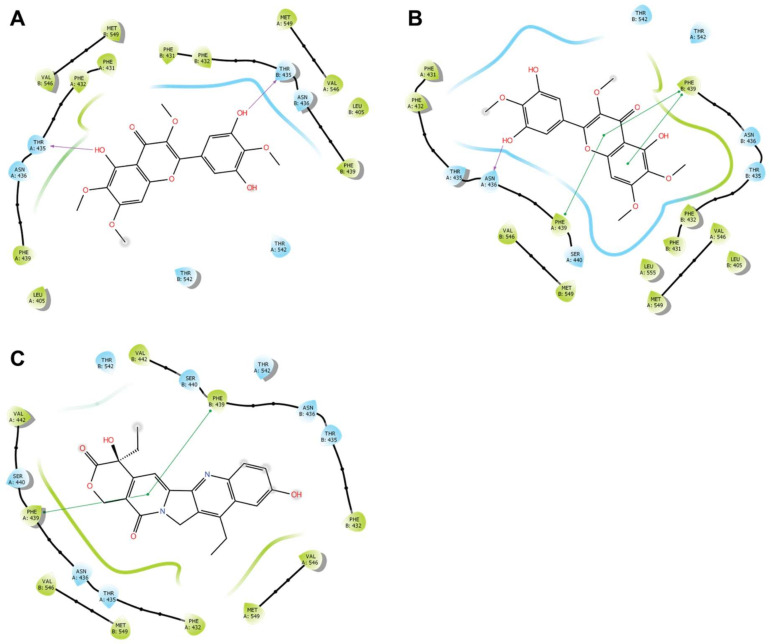
Overview of ligand interactions between ligand and ligand binding site in BCRP. Amino acid residues are coloured blue and green for hydrophilic and hydrophobic residues, respectively, and the surrounding of the ligand is likewise illustrated with blue and green lines. Hydrogen bonds are marked with purple lines, while π–π stacking hydrophobic interactions are marked with green lines. (**A**): Interactions between BCRP and docked **2**. (**B**): Interactions between BCRP and **2** after 600 ns MD simulation. (**C**): Interactions between BCRP and SN-38 as also seen in a previous study [28].

The docking experiments showed that compound **2** could be docked into the same binding site as found for the cryo-EM BCRP:SN-38 complex [28] (Figure 10) with a Glide docking score of −10.98 kcal/mol, which was comparable to the docking score of −12.24 kcal/mol reported for SN-38.

The docking shows that **2** forms hydrogen bonds between the hydroxyl group at the 5-position and the Thr435 residue in the A chain of BCRP (i.e., Thr435A) as well as between the hydroxyl group at the 5′-position and the Thr435 residue of the B chain (i.e., Thr435B) (Figure 10A). Noticeably, the experimentally determined structure of SN-38 in BCRP has no hydrogen bonding interactions but displays π–π stacking interactions with both Phe439A and Phe439B (Figure 10C), whereas compound **2** displays no π–π stacking interactions.

**Figure 11:**
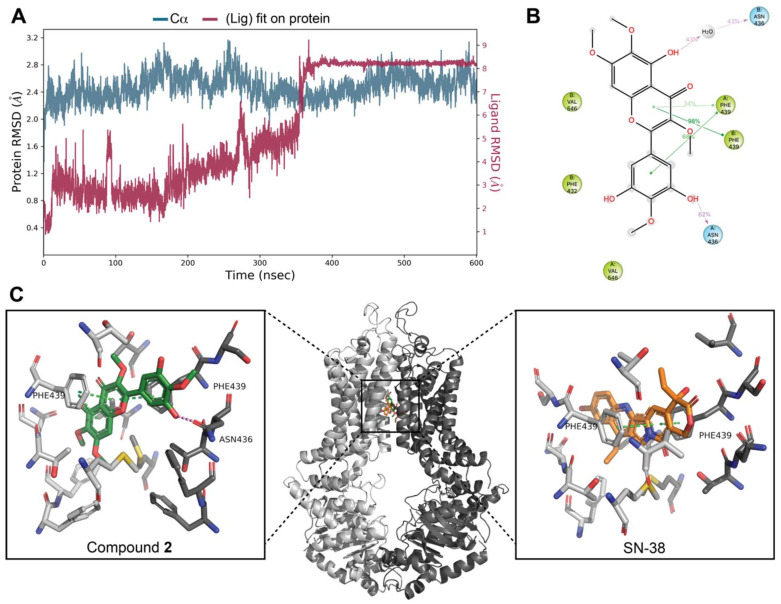
(**A**): Time trace of root mean square deviation (RMSD) of ligand position relative to that at the start of the MD simulation. (**B**): Details of interactions between BCRP and **2** during the 380–600 ns period of the MD simulation. Hydrogen bonds and water-bridged interactions are described by the violet lines, π–π stacking interactions are indicated by green lines and hydrophobic interactions are shown as grey spheres. Percentages indicate the fraction of nanoseconds where the interactions occur. (**C**): Binding site of BCRP (middle) with enlarged views of binding poses of **2** (left) and SN-38 (right) [28]. The active BCRP dimer consists of two monomeric units A (coloured light grey) and B (dark grey); compound **2** is coloured green, and SN-38 is coloured orange. Zoomed views show residues within 5 Å.

To further investigate the binding mode of **2**, 600 ns molecular dynamics simulations were performed with **2** docked in the BCRP transporter with water, ions, and a lipid layer added. The result immediately led to two conclusions. The ligand stayed within the binding cavity during the entire simulation. However, after 350 ns the ligand shifted pose dramatically by rotating approximately 135° around an axis orthogonal to the molecular plane (Figure 10B). The root mean square deviation (RMSD) time trace from the molecular dynamics experiment (Figure 11A), i.e., the RMSD of actual position relative to start position, shows that **2**, until approximately 350 ns (red line in Figure 11A), had average RMSDs similar to those seen for BCRP (blue line in Figure 11A). However, a dramatic change with significantly reduced movements of **2** occurred at 350 ns. The average interactions of **2** with BCRP obtained from the MD simulations from 380 to 600 ns, are shown in Figure 11B. Remarkably, at this point **2** had π–π stacking interactions with Phe439A and Phe439B (Figure 10B), similar to those observed for the BCRP:SN-38 cryo-EM structure (Figure 10C) [28]. Energy minimization of the BCRP structure after 600 ns and subsequent docking of **2** resulted in the same ligand pose as seen in the simulation after 350 ns and a Glide docking score of −11.67 kcal/mole.

## 4. Discussion

Efflux-pump-mediated MDR is a major obstacle for successful cancer treatment. Current efflux pump inhibitors have failed to reach the clinic due to undesired side effects, toxicity, and the poor clinical design of studies. Thus, there is a large unmet clinical need for new, improved drug candidates with fewer toxic effects. With a high chemical diversity, natural products constitute a promising source for new, fourth generation inhibitors. In the present study, we have shown that SPE fractions of a crude extract of *E. galeata* were able to increase the effect of SN-38 on HT29_SN38_ cells, and for the fraction eluted with 40% acetonitrile, an up to 41% increase in SN-38 cytotoxicity was observed. The fraction eluted with 50% acetonitrile also gave rise to some increase in activity, but it was also cytotoxic in itself, making it less interesting for further investigations regarding synergy with SN-38. Further work on the 40% fraction led to the isolation of 5,3′,5′-trihydroxy-3,6,7,4′-tetramethoxyflavone as the component responsible for a BCRP-inhibitory activity with an IC_50_ value of 14.67 µM and an ability to resensitize SN-38 resistant colon cancer cells. Furthermore, it was shown that this compound did not exhibit cytotoxic activity in itself, which is a prerequisite to be even considered a potential efflux pump inhibitor drug lead. The positive control, Ko143, exhibited an IC_50_ value of 0.029 µM, which is several-fold more potent than the isolated compound. However, it was seen from the results that the applied concentration of 5,3′,5′-trihydroxy-3,6,7,4′-tetramethoxyflavone was able to increase the effect of SN-38 to almost the same extent as Ko143 (Figure 6 and Figure 7), without any significant increase in cytotoxicity exerted by the compound alone. Therefore, even though the IC_50_ value was higher for compound **2**, the concentration needed to resensitize the cells to an extent comparable to that found for Ko143, compound **2** was not more cytotoxic at the concentration required for maximal synergy. 

Previous studies have shown that several classes of flavonoids are capable of inhibiting BCRP in vitro [33,34], and they are in general well-tolerated, since many dietary plants commonly consumed by humans contain various types of flavonoids. However, even though these compounds are generally non-toxic, their specificity towards BCRP is also important to assess. BCRP share structural similarities with other efflux pumps, such as P-glycoprotein and MRP1. All of these pumps are also expressed in normal human tissues, and are thus not restricted to cancer cells. It is therefore important for a potential BCRP inhibitor to exhibit specific inhibition, in order to avoid undesired inhibitory effects on other efflux pumps. Future studies should therefore investigate the specificity of 5,3′,5′-trihydroxy-3,6,7,4′-tetramethoxyflavone (**2**) for BCRP, to assess whether it could give rise to undesired effects in humans. 

Docking studies further confirmed that **2** occupies the same ligand binding site as SN-38. The pose identified after more than 350 ns of MD simulation is more likely to be correct than the pose from the initial docking. It has a slightly lower Glide docking score, stable interactions with BCRP and displays the same π–π stacking interactions as seen in the experimental BCRP:SN-38 structure. Hence, the computational studies suggest that compound **2** inhibits BCRP via competitive binding to the transporter substrate binding site with stacking of the aromatic ring system to Phe439A and Phe439B, which are very important interactions, analogous to what is observed in the binding pose of SN-38 (Figure 11C).

Since flavonoids have the same basic structure, with varying methoxylation, hydroxylation, and/or glycosylation patterns, differences in specificities are most likely affected or determined by these functional groups. The isolated flavone, 5,3′,5′-trihydroxy-3,6,7,4′-tetramethoxyflavone, has four methoxylations and three hydroxylations. It has previously been reported by Pick et al. [33] that methoxylation at position 3 and hydroxylation at position 5 are important for BCRP-inhibitory activity. The MD calculations confirm that hydroxylation at position 5 is important, as this group is engaged in water-mediated hydrogen bonding to Asn436B during 43% of the time course of the simulation. Furthermore, the methyl group of the methoxy group at position 3 has hydrophobic interactions with the transporter. Pick and coworkers [34] furthermore showed that hydroxylation at positions 2′, 3′ and 4′ decreased the BCRP-inhibitory activity, which may account for the IC_50_ of the isolated flavone being higher than that of Ko143, since the compound is hydroxylated at position 3′. The MD simulation suggests that the hydroxyl group at position 5′ forms a hydrogen bond to Asn436A during 62% of the simulation and thus contributes positively to the affinity. On the other hand, the hydroxyl group at position 3′ has unsatisfied hydrogen bonding possibilities, and therefore contributes negatively to the affinity. Taken together, the 3′ hydroxyl group contributes negatively to the affinity, in agreement with previous findings, and the 5′ hydroxyl group contributes positively to the affinity.

Only a few studies report in vitro assaying of 5,3′,5′-trihydroxy-3,6,7,4′-tetramethoxyflavone, and no NMR data or studies evaluating its efflux pump-inhibitory potential have been published. Therefore, we are reporting for the first time the NMR data and BCRP-inhibitory activity of this compound, and characterizing its ability to resensitize SN-38 resistant colon cancer cells toward SN-38 without significantly affecting cell viability by itself. The fact that this compound was isolated from a plant extract supports the notion that the vast pool of natural products may be an untapped source of fourth-generation efflux pump inhibitors. By now several flavonoid compounds have been found to possess promising BCRP-inhibitory activity, making them interesting as potential drug lead scaffolds.

## 5. Conclusions

An in vitro study was performed to evaluate the ability of constituents of *E. galeata* to reverse BCRP-mediated SN-38 resistance. Compound **2**, 5,3′,5′-trihydroxy-3,6,7,4′-tetramethoxyflavone, isolated from *E. galeata*, exhibited synergy with SN-38, which via a dye accumulation study was shown to be a result of BCRP inhibition. The molecular docking studies suggest that compound **2** binds in the substrate binding site of BCRP. Inhibition of the BCRP-mediated transport of SN-38 through competitive binding could therefore be a likely mode-of-action of **2**, but further experiments are needed to conclude this. 

*Eremophila* spp. are culturally important plants for many of Australia’s First Peoples, the Aboriginal peoples. If you use the information here provided to make commercial products, we urge you to strongly consider benefit sharing with the Aboriginal communities or groups in the areas where these species grow. We acknowledge that this work took place on the lands of Aboriginal peoples who are the custodians of this land and acknowledge and pay our respects to their Elders past and present.

## Figures and Tables

**Table 1:** ^1^H and ^13^C NMR data of **2**.

Structure	^1^H NMR δ (nH, m)	^13^C NMR δ
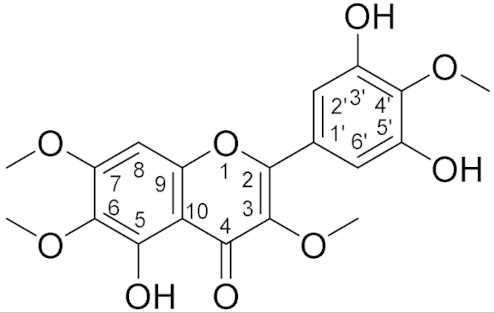	12.47 (1H, s, 5-OH), 7.31 (2H, s, H2′/H6′), 6.50 (1H, s, H8), 4.0 (3H, s, 4′-OMe), 3.96 (3H, s, 7-OMe), 3.92 (3H, s, 6-OMe), 3.88 (3H, s, 3-OMe)	154 (C-2), 139 (C-3), 178 (C-4), 152 (C5), 132 (C-6), 158 (C-7), 90.5 (C-8), 152.5 (C-9), 106.6 (C-10), 126.4 (C-1′), 108.9 (C-2′), 148 (C-3′), 136 (C-4′), 148 (C-5′), 108(C-6′), 61.4 (4′-OMe), 56.4 (7-OMe), 61.0 (6-OMe), 60.5 (3-OMe)
